# RETRACTION: Effect of Intramedullary Fixation and Plate Fixation on Postoperative Wound Complications in Clavicle Fractures: A Meta‐Analysis

**DOI:** 10.1111/iwj.70379

**Published:** 2025-03-21

## Abstract

**RETRACTION:** LinS. , HuangJ. , LaminB. M. , ZengT. , TianY. , LiuL. , and LuoH. , “Effect of Intramedullary Fixation and Plate Fixation on Postoperative Wound Complications in Clavicle Fractures: A Meta‐Analysis,” International Wound Journal 21, no. 1 (2024): e14361, 10.1111/iwj.14361.37641210 PMC10781614

The above article, published online on 28 August 2023, in Wiley Online Library (http://onlinelibrary.wiley.com/), has been retracted by agreement between the journal Editor in Chief, Professor Keith Harding; and John Wiley & Sons Ltd. Following an investigation by the publisher, all parties have concluded that this article was accepted solely on the basis of a compromised peer review process. The editors have therefore decided to retract the article. The authors disagree with the retraction.

**RETRACTION:** S. Lin , J. Huang , B. M. Lamin , T. Zeng , Y. Tian , L. Liu , and H. Luo , “Effect of Intramedullary Fixation and Plate Fixation on Postoperative Wound Complications in Clavicle Fractures: A Meta‐Analysis,” International Wound Journal 21, no. 1 (2024): e14361, 10.1111/iwj.14361.37641210 PMC10781614

The above article, published online on 28 August 2023, in Wiley Online Library (http://onlinelibrary.wiley.com/), has been retracted by agreement between the journal Editor in Chief, Professor Keith Harding; and John Wiley & Sons Ltd. Following an investigation by the publisher, all parties have concluded that this article was accepted solely on the basis of a compromised peer review process. The editors have therefore decided to retract the article. The authors disagree with the retraction.

